# Antibiotic use and resistance: an unprecedented assessment of university students’ knowledge, attitude and practices (KAP) in Lebanon

**DOI:** 10.1186/s12889-020-08676-8

**Published:** 2020-04-19

**Authors:** Samer Sakr, Ali Ghaddar, Bassam Hamam, Imtithal Sheet

**Affiliations:** 1 Department of Biological and Chemical Sciences, School of Arts and Sciences, Lebanese International University, Beirut, Lebanon; 2 Department of Biomedical Sciences, School of Arts and Sciences, Lebanese International University, Beirut, Lebanon

**Keywords:** Antibiotic resistance, Antibiotic use, Attitude, Knowledge, Practice, KAP, Health related majors, And non-health related majors

## Abstract

**Background:**

The emergence and spread of pathogenic bacteria that is resistant to antibiotics has become a major public health concern. The incorrect prescription, inappropriate consumption and excess use of antimicrobial drugs, specifically antibiotics, are possibly the main factors contributing to the widespread of antibiotic resistant bacteria. This study aims to evaluate the knowledge, attitude and practices (KAP) towards the use of antibiotics as well as their resistance among Lebanese university students in health and non-health related majors.

**Methods:**

This cross-sectional study was conducted between May and June 2019 in Beirut (Lebanon) in which 750 students completed a questionnaire made up of four dimensions: Socio-demographic characteristics, 3 questions; assessment of knowledge, attitude and practices, 7, 10 and 1 question, respectively. The data was collected in spreadsheets and analysed with descriptive statistics. The difference in mean scores in each of the knowledge, attitude and practices dimensions between health and non-health related major students was analysed using t-student tests and the difference in percentages using chi-square tests.

**Results:**

Almost 78% of respondents from the health related majors scored high knowledge compared to only 41% of non-health related majors (mean = 4.26; standard error = 0.05 versus mean = 3.41; standard error = 0.13, respectively). The attitude score of the health related major students (35.42%) was positive and more satisfactory compared to the non-health related students (7.32%); (mean = 9.34; standard error = 0.05 versus mean = 9.10; standard error = 0.21, respectively). However, the difference in the scores of attitudes was not statistically significant.

**Conclusions:**

Interventions to promote awareness in this area should focus more students in on non-health related majors.

## Background

Over the last 5 decades, antibiotics have proven to be an effective and decisive weapon against several diseases. Today, the emergence of pathogenic bacteria that have become resistant to antibiotics, and their spread in the human population, is a growing problem worldwide presenting a significant threat to public health in the twenty-first century, particularly in the developing countries [1, 2]. Self-medication, incorrect prescription, inappropriate consumption and excessive use of these antimicrobial drugs could be the key factors for the increase and spread of antimicrobial resistance (AMR) in addition to other equally important social and cultural factors [2–5]. This increase in antibiotic resistance will eventually diminish their therapeutic effectiveness and increase treatment failures leading to more severe illnesses with higher mortality rates [6]. Not to mention the heavy burden this will have on the global economies as well as the different healthcare management systems [7].

Antibiotic self-medication has become a serious concern and a leading cause of antibiotic resistance. Antibiotic self-medication can result from many factors, such as poor public knowledge and attitude towards antibiotics, easy access to antibiotics in many places and lack of awareness policies on appropriate antibiotic usage [8]. Numerous studies have reported improper antibiotic use among university students due to self-medication and lack of adequate knowledge of antibacterial agents. Specifically, their indications, their specificity to pathogens and the compliance to dosage regiments [9].

The assessment of knowledge, attitude and practices (KAP) on a representative sample of university students could be an efficient tool to help improve the use of antibiotics [10]. Jairoun and colleagues (2019), after conducting a KAP study related to the use of antibiotics among university students in the United Arab Emirates, recommended in their conclusions the development of comprehensive programs and effective educational interventions to remediate the gap in the medical curriculum leading to self-medication practice [8]. In the same year, Al-Salih and colleagues, after conducting a study about knowledge and attitude regarding antibiotic use among nursing and dentistry students in Babylon University (Iraq), have concluded that despite their knowledge about the appropriate antibiotic use, students lacked in the appropriate attitude [11]. The same conclusion has been reached regarding professional Indian medical students by Khajuria and colleagues in 2018 [12]. A cross-sectional questionnaire based study conducted among 2500 Chinese students regarding their KAP of antibiotics concluded that the medical curriculum improves the students’ knowledge on antibiotics. However, since senior medical students showed excessive use of antibiotics, this indicated a lack of appropriate instructions on antibiotic use in their curriculum [13]. The attitude toward antibiotic use and resistance was average among students of International university of Africa (Sudan) despite having good knowledge as reported by Sunusi and colleagues in 2019 [9].

Chamoun et al. have reported in 2016, based on a study about the prevalence of antibiotic resistance in 76,278 isolated bacterial strains obtained from 16 Lebanese hospitals (between the years 2013 and 2016), that antimicrobial resistance is becoming a major problem in Lebanon [14]. In 2017, a study conducted by El Khoury and collaborators, concluded that the low educational and socioeconomic levels of parents as factors significantly associated with poor knowledge and misuse regarding antibiotics [15]. Cheaito and colleagues, in 2014, conducted a survey among buyers of antibiotics in pharmacies. Their results show that 42% of the participants reported purchasing antibiotics without a prescription. Whereas, almost 19% of the respondents, declared referring to the advice of the pharmacist. Almost 40% justified these practices as a way to save money [16].

To our knowledge, the evaluation of KAP about rational use of antibiotics among university students, enrolled into health related majors and non-health related majors, in Lebanon has not been assessed yet. Accordingly, the current study aims to evaluate the knowledge, attitude and practices (KAP) towards the antibiotic use and resistance among the university students with health and non-health related majors.

## Methods

### Study design, procedure and sample size

The current study relies on a cross-sectional questionnaire-based survey conducted among a random sample of students enrolled in the largest private university in Lebanon attended mostly by the middle income population ‘see Additional file 1 for the Questionnaire’. The study population comprises of a total of 1250 students. A simple random sample was taken to include students with all health-related majors (biology, biochemistry, medical laboratory, food science, nutrition and pharmacy) as one group, and non-health-related majors (business, engineering, education, arts and computer sciences) as another group.

### Data collection

This cross-sectional survey was conducted during the period between May and June 2019. A structured questionnaire was designed and developed by the research team based on literature review and was adapted to cover all the main key points of the research topic (antibiotic use and resistance). The adopted questions were mainly based on previous studies [7, 8, 17–19] and were slightly adapted to the context of the conditions in Lebanon. A pilot study was conducted among 12 students to assess the reliability and validity of the instrument. Data from the pilot study was excluded from the results, but served for adjusting minor modifications to the questions based on the analysis of the collected comments. The reliability of the questionnaire was assessed by calculating the alpha-Cronbach’s coefficient which were found to be satisfactory for the three dimensions of the questionnaire (knowledge: alpha-Cronbach = 0.68, attitudes: alpha-Cronbach = 0.76 and practices: alpha-Cronbach = 0.71). An electronic link (Google form) of the questionnaire was emailed to approximately 1250 students from different majors who were also encouraged to fill the questionnaire during class sessions by their instructors in order to reduce the information and selection bias. Seven hundred fifty students responded to the questionnaire (response rate = 60%), out of which, 63.60% (*n* = 477) were majoring in one of the following health related majors: biology, biochemistry, nutrition, food sciences, biomedical sciences and pharmacy. The remaining 36.4% of students (*n* = 273) were in non-health related majors such as: business administration, arts, engineering or education.

### Variables

The questionnaire included dimensions on the KAP (knowledge, attitude and practices) towards antibiotic use and resistance. The data was collected in excel sheet and analysed with descriptive statistics and results expressed as means and standard deviations, frequencies and percentages. Questions were grouped into four categories reflecting the participants’ socio-demographic characteristics (3 questions including age, gender and education), knowledge (7 questions including, as an example, “Are antibiotics effective to treat urinary tract infection?”), attitude (10 questions including, as an example, Is it okay to buy the same antibiotics, if you are sick and they helped you get better and practices (1 question, “Do you check the expiry date of the antibiotic before using it?”). Participants scored 0 on each question with the wrong answer and 1 for each question with the right answer. The sum of scores was calculated for the two dimensions of knowledge and practices considering the sum of the score for each individual question in each dimension. Scores ranged from 0 to 7 in the knowledge dimension and from 0 to 10 in the attitudes dimension.

### Statistical analysis

Descriptive statistics showed the frequency and percentage (%) of participants who answered correctly for the different questions related to socio-demographic characteristics, knowledge, attitude and practices towards antibiotics use. Chi-square test of independence was used to compare frequency of participants who answered correctly between the health and non-health related majors. T-student test was used to compare the average score in the two domains of knowledge and attitude between students in the health and non-health related majors.

## Results

### Study participants

A total of 1250 participants was randomly selected out of 10,000 students enrolled at the university. Seven hundred fifty students (out of 1250) responded to the questionnaire giving this study a response rate of 60%. Respondents were categorized into two groups based on their respective majors: 63.60% (*n* = 477) were majoring one of the following health related topics (biology, biochemistry, nutrition, food sciences, biomedical sciences and pharmacy); while the remaining students 36.4% (*n* = 273) were studying a non-health related major (business administration, arts, engineering or education).

### Socio-demographic characteristics of participants

As indicated in Table 1, more than half of the participants 568 (75.73%) were females and 182 (24.26%) were males. The majority of the participants 493(65.73%) were aged 18–21 years; and 161 (21.46%) participants were aged 22–23 years; 95 (12.66%) and one (0.13%) participants were aged more than 23 and less than 18, respectively. The majority of participants 477 (63.60%) were enrolled in health related education and 273 (36.40%) were in non-health related education.

**Table 1:** Socio-demographic characteristics of respondents/participants (*n* = 750)

Socio-demographic characteristics	Number (Frequency %)
*Age*	
18–21	493 (65.73)
22–23	161 (21.46)
> 23	95 (12.66)
< 18	1 (0.13)
*Gender*	
Female	568 (75.73)
Male	182 (24.26)
*Education*	
Health Related Majors	477 (63.60)
Non-Health Related Majors	273 (36.40)

### Knowledge of antibiotics use among participants

Table 2 indicates the frequency and % of participants who answered yes/no for each question related to knowledge, attitude and practices towards antibiotics use. The % of students in health-related majors who got the correct answer was higher than those in non-health related majors for the majority of questions related to knowledge (effectiveness of antibiotics for treating viral vs. bacterial infections, for treating urinary tract infections, for treating fever, the right time to stop taking antibiotics and the familiarity with terms related to antibiotic resistance). These differences in scores were statistically significant in most of the questions except for the question related to effectiveness of antibiotics for the treatment of fever. On the other hand, the % of students in non-health related majors who answered correctly was higher in only two questions related to effectiveness of antibiotics to treat malaria and headaches; however, the difference was statistically significant only for the question related to malaria. The biggest difference in the answers of students from the two groups was in the question about effectiveness of antibiotics to treat viral infections, where 80.2% of students in health related majors gave the correct answer compared to only 36.9% of students in the non-health related majors. It is interesting to note that the majority of students in both groups knew when to stop using antibiotics (95 and 80.7% in the health and non-health related majors, respectively). The percentages of participants who got the correct answers in both groups, together with the *p*-values are also showed in Table 2.

**Table 2:** Number and percentage of the correctly answered questions related to KAP in health and non-health related majors

	Number (Percentage) of Respondents Giving a Correct Answer		
	Non-health related major	Health-related major	*p*-value
***Knowledge***	n (%)	n (%)	
Effectiveness of antibiotics to treat bacterial/viral infections	101 (36.9)	381 (80.2)	≤0.001
Effectiveness of antibiotics to treat urinary tract infections	51 (30.4)	215 (47.3)	≤0.001
Effectiveness of antibiotics to treat malaria	88 (57.5)	208 (53.3)	0.38
Effectiveness of antibiotics to treat headaches	41 (17.7)	54 (11.6)	0.02
Effectiveness of antibiotics to treat fevers	84 (37.2)	188 (40.3)	0.43
When should you stop taking antibiotics once you had started the treatment?	221 (80.7)	452 (95.2)	≤0.001
Familiarity with terms related to antibiotic resistance	155 (56.6)	443 (93.3)	≤0.001
***Attitude***			
Is it okay to use antibiotics that were given to a friend or family member, as long as they were used to treat the same infection as you have?	202 (85.2)	435 (94)	≤0.001
Is it okay to buy the same antibiotics, if you’re sick and they helped you get better when you had the same symptoms before?	163 (68.2)	384 (84)	≤0.001
What do you think of a doctor who doesn’t prescribe antibiotics when the patient thinks that they are needed?	120 (68.6)	305 (83.1)	≤0.001
Does insufficient knowledge contribute to the development of antibiotic resistance?	117 (84.7)	398 (89.4)	0.21
Do you think that antibiotic resistance can result from inappropriate use of antibiotics?	158 (89.2)	425 (94.4)	0.17
Does skipping one or two doses of antibiotics contribute to the development of antibiotic resistance?	101 (70.6)	334 (82.3)	0.003
Do you think that the more antibiotics we use in society, the higher is the risk that resistance develops and spreads?	143 (83.6)	421 (90.5)	0.01
Can antibiotic resistance spread from one person to another?	224 (81.8)	357 (75.2)	0.07
Is antibiotic resistance an important and serious global public health issue?	164 (83.6)	441 (94.6)	0.03
Should pharmaceutical companies, in your opinion, develop new antibiotics?	89 (89.6)	306 (98.6)	0.04
***Practices***			
Do you check the expiry date of the antibiotics before using it?	204 (74.5)	401 (84.4)	0.001

### Attitude towards antibiotics use among participants

The % of participants who gave correct answers in the health related majors was higher than the non-health group on all questions related to attitude except for the question about attitude towards the spread of antibiotic resistance from one person to another where the non-health group scored higher with *p*-value = 0.07. The difference in scores between the health and the non-health group was statistically significant in all of the questions except for two questions about attitudes towards antibiotic resistance spread due to insufficient knowledge and inappropriate use (*p* = 0.21 and 0.17, respectively). The % difference of participants who had the correct attitudes towards antibiotic resistance was the highest in the question about buying the same antibiotics that helped treat the same symptoms in the past (84% vs. 68.2% for health and non-health groups respectively).

Participants were also asked about their attitude whether or not pharmaceutical companies should develop new antibiotics. The % of respondents who believed that pharmaceutical companies should develop new antibiotics was higher in the health-majored students (98.6% vs. 89.6%). A rapid qualitative analysis of the participants’ written responses while answering one question about their subject opinion on the subject matter revealed that both groups (health and non-health) were aware about the importance of developing new antibiotics to deal with issues of resistance. It was noted that some non-health majoring students were aware that “new viruses and infections are born and most cannot be properly treated with existing antibiotics” as given by this example. While, most health related students used more scientific terms to express their opinions as in this example: “Because the bacterial cells will be more resistant to the current antibiotics due to mutations that occur.”

### Practices on antibiotics use among participants

When asked about previous use of antibiotics, 50% of all participants answered that they have previously used Augmentin. The second and third most commonly used antibiotics were Flagyl (12%) and Amoxicillin (9%). 10% answered that they have never used antibiotics before.

Practices were assessed through asking participants whether they check the expiry date of the antibiotic before using it. The majority of participants in both groups (84.4% in health and 74.5% in non-health related majors replied that they do check. The % was higher in the health-related major group with statistically significant difference form the non-health group (*p*-value≤0.001).

### Analysis of overall knowledge and attitude scores

The average knowledge score was higher in the health major group of students compared to the non-health group (mean = 4.26; standard error = 0.05 vs. mean = 3.41; standard error = 0.13, respectively). This difference in scores was statistically significant (*p*-value≤0.001). The average attitude score was higher in the health major group of students compared to the non-health group (mean = 9.34; standard error = 0.05 vs. mean = 9.10; standard error = 0.21, respectively). However, the difference in the scores of attitudes was not statistically significant (*p*-value = 0.12).

## Discussion

Antibiotic resistance is a serious public health problem. Assessment of knowledge, attitude and practices of antibiotic use in university students can greatly impact how best to tackle the growing threat of the antibiotic resistance and its related issues [18, 20]. In the present study, students in health related majors had better knowledge (higher percentages of correct answers) in almost all the questions related to knowledge; and, had more informed attitude towards dealing with the problem of antibiotic resistance (had higher percentages of correct answers in all questions related to attitude). Thus exhibiting a good knowledge, and satisfactory behavioural attitude towards a rational use of antibiotics. The knowledge and attitude of students in non-health majors were, as expected, less satisfactory. Strikingly, both groups of students have shown exemplary attitude when it comes to antibiotic resistance. As for practices, most of the students regardless of their majors were well aware of good practices. Our findings showed that 80.2% (*n* = 381) of students in health related majors were aware that antibiotics are used to treat bacterial infections. Whereas 36.9% (*n* = 101) of the students in non-health related majors were knowledgeable about the effectiveness of antibiotic against bacterial or viral infection. Moreover, the current study revealed that the large majority of students in health related majors compared to non-health majors (95.2% vs. 80.7, respectively), were knowledgeable about the timing of when to stop the antibiotics use.

In an article published in 2017 by Jamhour et al. including 400 adults’ respondents from two cities in Lebanon, they found that 61% thought that antibiotics should be taken as a common cold treatment. They also showed a significant correlation between self-medication and lower educational level. In addition, the respondents in that study who had lower knowledge about antibiotics, usually stopped antibiotics at the inappropriate time [21]. Mouhieddine and colleagues have reported in 2015, based on a random convenience sample of 500 people in Lebanon, that 46.1% of them expressed moderate knowledge levels, where 3.5% did not know that antibiotics are not anti-viral. In this study, 56.0% of the respondents also expect the doctor to prescribe an antibiotic for the common cold [22]. Similarly, Jifar and Ayele in 2018, reported that 83% of respondents in Harar city, Eastern of Ethiopia, replied that antibiotics speed up the recovery colds [17]. On the other hand, Jairoun and his colleagues have reported in 2019 that the large majority of university students were aware that antibiotics can kill bacteria and can be used to cure bacterial infections [8]. Moreover, Khajuria et al. 2019 showed that 90% of medical students agreed that antibiotics are useful for bacterial infections [12]. Gary and colleagues, have reported in 2012, while comparing the KAP related to antibiotic use and resistance among medical and non-medical university students in Jordan, that 44% percent of non-medical students and 28.1% of medical students agreed that antibiotics could cure cold and viral infections [23]. In Britain, 38% of respondents ignored that antibiotics cannot resolve colds [24]. Several studies have revealed that antibiotics are more likely to be prescribed under patients’ pressure [25]. Another study revealed widespread misconceptions about the utility of antibiotics for viral infections [26]. This is consistent with the findings of a global survey conducted by the world health organization (WHO) in 2015 [27]. WHO established a key strategy by engaging the prescribers and educating the public to reduce misuse of antibiotic use [28, 29].

In our study, the majority of the students enrolled in health related majors (93.3%), were familiar with terms related to antibiotic resistance, whereas around half of non-health related major students (56.6%) were aware of such terms. Jamhour and colleagues published in 2017 that 83% of the 500 respondents in Lebanon knew that the misuse of antibiotics could result in microbial resistance [21]. In a report published in 2015, it showed that 48.5% of respondents from Lebanon, declared continuing to take their full course of antibiotics even if their symptoms improved, underlying an alarmingly 51.5% who could stop their treatment after symptoms improvement [22]; similar to what was found by a number of previous studies [22, 30, 31].

In the current study, about half of the university students reported the use of antibiotics at least once in the year prior to study. A study done in Lebanon (2015) showed that 68.3% of the considered sample consumed antibiotics 1–3 times per year [22]. Our data is more consistent with a study (53.5%) conducted in Harar city, Eastern Ethiopia [17]. At the same time, our scores were higher than what was reported by Tesfaye (2017) who reported that 35.9% of participants consume antibiotics once during the year preceding the study [32]. On the other hand, our finding was considerably lower than what was found in Namibia, which was 80% [33].

The average knowledge score was significantly higher in the health major group compared to the non-health major students (*p*-value≤0.001). The average attitudes score was higher in the health major group of students compared to the non-health group. When comparing our data with other studies conducted among university students, our results agree with the survey conducted among medical and non-medical Chinese university students as reported by Huang et al. 2013 where they reported that Medical students were better than non-medical students in terms of attitude, knowledge and perception on the level of public education on antibiotic use, but worse on behavior. However, they found that but senior medical students have more positive behavior on the usage of antibiotics compared with low grade medical students and non-medical students in general [13]. Same pattern was observed by another study of medical school students scoring remarkably better than those the non-medical school in KAP towards antibiotic use and resistance [8]. In another study, it was found that 80% of nursing and dentistry students in Babylon University, Iraq have high knowledge but inappropriate attitude [11], results which were similarly found among Indian and Sudanese medical university students [9, 14]. Higuita-Gutiérrez and colleagues, reported in 2020 that medical students from three medical schools in Medellin, Colombia exhibit poor knowledge regarding antibiotic use due to insufficient training with regard to antibiotic use and bacterial resistance [34]. Whereas, Veses and colleagues in the same year, after surveying undergraduate dental students at Universidad Cardenal Herrera, concluded that awareness campaigns are needed to promote student’s use of antibiotics in young generations particularly among the pre-professional health sciences students [35]. Interestingly, as a result of the lack of training they discovered, Tsopra et al. 2020 used a game called ‘AntibioGame’ through which students play the role of a doctor meeting patients in consultation as a promising tool for improving knowledge in antibiotic prescription [36].

Taking all of the above into consideration, as well as our findings about the lack of appropriate knowledge among university students, irrespective of their major, when questioned about the effectiveness of antibiotics to treat urinary tract infection, malaria, and headache or fever (where the correct answers varied between 17.7 and 53.3%), we recommend that awareness programs and educative measures must be better incorporated in students’ curricula to remediate the gaps related to their knowledge about antibiotic use.

As for the attitude assessment, all students, whether they were enrolled in health or non-health related majors, agreed that AMR is a serious public health issue and that repeated use of antibiotics and insufficient knowledge could lead to antibiotic resistance. These findings were similar, though in better numbers, to the previous studies [20, 31]. Jifar and Ayele in 2018, published in their study that 78.4% of subjects in Harar city agreed that the unnecessarily use of antibiotics can increase the antibiotic resistance [17], 69.7% in Ethiopia [32], 50% in Jordan [37], and 72% in Namibia [33]. Our presented results revealed that students with health related major had a favorable and better attitude about rational use of antibiotics compared to the group of students with non-health major. Only 68.6% (*n* = 120) of the students with non-health major agreed that “a doctor is a good one even if he does not prescribe antibiotics when the patient thinks that it is needed” whereas, 83.1% (*n* = 305) of the opposite group of students share the same opinion. It has been published by Mouhieddine and his colleagues that 65.1% of the 500 respondents questioned in Lebanon in 2015, referred to doctors’ prescriptions regarding antibiotics, and 22.4% declared, alarmingly, that they self-medicate [22]. In the study of Jifar and Ayele in 2018, most respondent (90%) agreed on the need for physician consultation before purchasing antibiotics and 73.1% declared getting prescription to purchase antibiotics. This finding is just higher than study done in Saudi Arabia in which they reported, 76.6 and 66.6%, respectively for the same questions [17]. According to Jamhour and colleagues in 2017, it is common that Lebanese get access to antibiotics without a prescription despite the high ratio of physicians to patients in Lebanon [38]. The same pattern was observed in our study when students of different majors where questioned about the efficiency of using same antibiotics to treat same symptoms faced in a previous disease. Almost 70% of non-health related major compared to 84% of students of the second group answered this question correctly. Surprisingly, significant number of both group of students, 85.2% (*n* = 202) and 94% (*n* = 435) from non-health and health majoring students respectively, found it unacceptable to use antibiotics from a friend or family member to treat an infection. On the same topic, it has been reported by Jifar and Ayele in 2018 that 87.2% of respondents in Harar city in Ethiopia were aware not to keep antibiotics for future use; 90% also thought that antibiotics should not be shared among friends and family members without prior physician consultation; and 65.3% self-prescribed antibiotics translating poor knowledge and attitude toward antibiotics use [17]. Another study reported that only 17% of participants kept antibiotics in their home for future use [39]. A Namibian study reported that 28.5% of users kept antibiotics for future use [40]. In India, 76% used antibiotics without prescription [41], 32.7% in Italy [42], 28.8% in Saudi Arabia [43], and 9% in Hong Kong [44]. This difference might be due to variation of regulations and their application from one country to another in addition to differences in the socio-demographical conditions. The findings presented in the current study indicate that students were not well aware of the irrational use of antibiotics, though students in health majors showed a better attitude, this is different from findings published previously [45, 46]. Based on our sample, university students were well aware of the development of antibiotic resistance. However, the responses of the students in the present study cannot be generalized to other universities, since students could have different educational programs, skills and experiences [47, 48]. It is indeed a striking findings of this study having such a disparity in the attitude towards antibiotic use where 100% of students, regardless their respective majors, are aware of the issue of antibiotic resistance but up to 32%, in some cases, lack the appropriate attitude, particularly among the group of students in non-health related major. In this regards, our findings are similar to those reported by Mouhieddine et al., in 2015 that 40.6% of the 500 respondents demonstrated only moderate attitudes [22].

Our findings clearly indicate that it is urgent to limit the granted access for antibiotics in Lebanon and other developed countries. Indeed, the WHO is voicing alarms about the increasing levels of the development, worldwide, of antibiotic resistant pathogenic bacteria. In order to remediate to this major issue, the WHO issued a “Global Strategy for Containment of Antimicrobial Resistance” pressing governments and decision makers to apply and take actions as has happened in South Korea where a number of national educational campaigns on the appropriate use of antibiotics in various ways targeting the general public have been implemented [49]. Our report shows that knowledge and attitude, of university students, towards antibiotic use and antibiotic resistance could be positively impacted, though not always sufficiently, by more specialized course material related to health. This strengthens the need of the inclusion in the curriculum of students in non-health majors of strategies allowing to get familiarized with public health issues.

Our study highlighted the possible need for knowledge-based education programs for students, especially in the non-medical or non-health related fields. Specifically, our suggestions include seminars, workshops and courses in students’ curricula the extent and effectiveness of which can be the aim of future studies. The quick implementation of awareness campaigns about knowledge and appropriate use of antibiotics seems to be a priority based on ours and others findings. In addition, health authorities should expand their investments in policy making and in a more rigorous surveillance system regarding the access to antibiotics. Awareness campaigns could be done in a number of different routes: i) through national strategies promoting vaccination and hygiene; ii) by updating curricula in universities including public health courses/ workshops in all majors; ii) through media campaigns and intervention; and, iv) through a greater proactive role for pharmacists. The absence of such strategies could result in a continuous degradation of the KAP towards antibiotic use and resistance, leading to more serious consequences on the development of AR.

### Limitations of the study

The present study has a few limitations. To start with, results should be treated with caution before their generalization to the population of university students in Lebanon or the region since they are based on a cross-sectional design among a random sample of one university in Lebanon. Although the response rate was acceptable (60%), further studies with larger sample sizes and including more university and non-university students are needed to understand better the level of awareness of young adults and adolescents about the issue of antimicrobial resistance in Lebanon and the region.

## Conclusions

Antimicrobial resistance is a serious public health problem. Assessment of knowledge, attitude and practices of antibiotic use in university students can greatly impact the antibiotic related issues. This study addressed KAP about use of antibiotics and AR among students enrolled in a large private university in Lebanon. Our findings indicate that improving the students’ level of knowledge about the use of antibiotics might remediate and rationalize their attitude toward antimicrobial use. The curriculum of students with non-health related majors requires improvement to include seminars, workshops, and/or courses related to public health concerns such as AMR. Our recommendations are in line with what has been proposed by several comparable studies [7, 8, 13]. This would increase the knowledge of students with non-health majors towards public health issues. Awareness campaigns through media considering public health is also recommended. Surveillance system restricting the granted accessibility of antibiotics is an urgent need added to the involvement of the clinicians in sharing more efficiently their knowledge with the patients would aid in ensuring rational use of antibiotics and thus control the growing problem of antibiotic resistance. Involving the civil society organisation and the media intervention would greatly serve this aim.

## Supplementary information

**Additional file 1.** Questionnaire form.

## Data Availability

The datasets generated and/or analyzed during the current study are available from the corresponding author on reasonable request.

## Abbreviations

AMR: Antimicrobial resistance
KAP: Knowledge, attitude and practices
AR: Antibiotic resistance
WHO: World Health Organization
