# Supplementary material for: Antibiotic use and resistance: an unprecedented assessment of university students’ knowledge, attitude and practices (KAP) in Lebanon
Source: BMC Public Health. 2020 Apr 19;20:535. doi: 10.1186/s12889-020-08676-8 (PMC7169022; doi:10.1186/s12889-020-08676-8)
Supplement: Supplementary file 1 — Additional file 1. Questionnaire form. [file 12889_2020_8676_MOESM1_ESM.pdf]

Antibiotic use, prescription, knowledge, attitude and resistance

This questionnaire aims to assess the knowledge of students at the Lebanese International University about the use, advantages and disadvantages of antibiotics. All the information will stay unanimous and your participation is voluntary. There is no penalty for not participating and there is no reward for doing so. Also, you can withdraw at any time.

* Required

Sociodemographic characteristics

1. **What is your age? ***

Mark only one oval.

- 18-21 years old
- 22-23 years old
- More than 23 years old
- Less than 18 years old

2. **What is your gender? ***

Mark only one oval.

- Male
- Female

3. **Please select your major: ***

Mark only one oval.

- Engineering
- Business
- Pharmacy/Pre-pharmacy
- Education
- Arts
- Math/Physics/Chemistry
- Computer Science/Information Technology
- Biology/Biochemistry/Nutrition/FDSC
- Biomedical Sciences

4. **Your year of study** *
Mark only one oval.

- First year
- Second year
- Third year
- Other: ______________

Infections

This part aims to assess the knowledge about infectious diseases

5. **Have you ever had any of these infections?** *
Mark only one oval.

- Gatrointestinal tract infection
- Respiratory tract infection
- Urinary tract infection
- I had no infection
- I cannot remember
- Other: ______________

6. **Was it a viral or bacterial infection?** *
Mark only one oval.

- Viral infection
- Bacterial infection
- I do not know

Knowledge about antibiotics

This part aims to assess to which extent you are familiar with the antibiotics and their efficiency

7. **Are antibiotics effective against?** *
Mark only one oval.

- Bacteria
- Viruses
- Both
- I do not know

8. **Please indicate which of the following antibiotics you have used (most frequently) to treat infection?** *
Mark only one oval.

- Augmentin
- Klacid
- Neuroxin
- Flagyl
- Amoxicillin
- Zithromax
- Ciprofloxacin
- Ceftriaxone
- I do not remember the name of antiobiotics
- I have never taken antibiotics

9. **When was the last time you took antibiotics?** *
Mark only one oval.

- 2 years ago
- I do not remember when was the last time I took antibiotics
- I have never taken antibiotics
- During the last year

10. **Have you recovered from the infection after antibiotic use?** *
Mark only one oval.

- Yes
- No, I had to take another antibiotic in order to recover

11. **Do you think that urinary tract infection (UTI) can be treated with antibiotics?** *
Mark only one oval.

- Yes
- No
- In some cases
- I do not know

12. **Do you think that Malaria can be treated with antibiotics?** *
Mark only one oval.

- Yes
- No
- In some cases
- I do not know

13. **Do you think that Headache can be treated with antibiotics?** *
Mark only one oval.

- Yes
- No
- In some cases
- I do not know

14. **Do you think that Fever can be treated with antibiotics?** *
Mark only one oval.

- Yes
- No
- In some cases
- I do not know

15. **When should you stop taking antibiotics once you had started the treatment?** *
Mark only one oval.

- Immediately after feeling better
- After finishing all the pills as recommended by your doctor
- I do not know

Assessment of the antibiotic use

This part aims to assess the accessibility and way of antibiotic use

16. **Is it okay to use antibiotics that were given to a friend or family member, as long as they were used to treat the same infection as you have?** *
Mark only one oval.

- Yes
- No
- I do not know

17. **Is it okay to buy the same antibiotics, if you're sick and they helped you get better when you had the same symptoms before?** *
Mark only one oval.

- Yes
- No
- I do not know

18. **Do you need a prescription to get antibiotics from pharmacies in Lebanon?** *
Mark only one oval.

- Yes
- No
- I do not know

19. **Do you check the expiry date of the antibiotic before using it?** *

Mark only one oval.

- ( ) Yes
- ( ) No
- ( ) Sometimes

20. **Do pharmacists take their time to inform you on how antibiotics should be used?** *

Mark only one oval.

- ( ) Yes
- ( ) No
- ( ) Sometimes

21. **What do you think of a doctor who doesn't prescribe antibiotics when the patient thinks that they are needed?** *

Mark only one oval.

- ( ) A good doctor
- ( ) Not a good doctor
- ( ) I do not know

Antibiotic Resistance

This part aims to assess your knowledge about microbial resistance against antibiotics

22. **Have you heard of any of the following terms? I have heard of:** *

Check all that apply.

- [ ] Antibiotic resistance
- [ ] Superbugs
- [ ] Bacterial resistance against antibiotics
- [ ] Drug resistance
- [ ] None of them

23. **Do you think that antibiotic resistance is the result of insufficient knowledge about antibiotic use?** *

Mark only one oval.

- ( ) Yes
- ( ) No
- ( ) I do not know

24. **Do you think that antibiotic resistance can result from inappropriate use of antibiotics outside the doctor's prescription?** *

Mark only one oval.

- ( ) Yes
- ( ) No
- ( ) I do not know

25. **Does skipping one or two doses of antibiotics....** *

Mark only one oval.

- Contribute to the development of antibiotic resistance
- Do not contribute to the development of antibiotic resistance
- I do not know

26. **Do you think that more antibiotic we use in society, the higher is the risk that resistance develops and spreads?** *

Mark only one oval.

- Yes
- No
- I do not know

27. **Can antibiotic resistance spread from one person to another?** *

Mark only one oval.

- Yes
- No
- In some cases

28. **Today, is antibiotic resistance an important and serious global public health issue?** *

Mark only one oval.

- Yes
- No
- I do not know

Perspectives

It aims to have your feedback about the need of new antibiotics in Lebanon

29. **Should pharmaceutical companies, in your opinion, develop new antibiotics?** *

Mark only one oval.

- Yes
- No
- I don't know

30. **If your answer to the above question is YES, explain why it's important to develop new antibiotics?**

____

Powered by
Google Forms
